# Direct Activation of TRPC3 Channels by the Antimalarial Agent Artemisinin

**DOI:** 10.3390/cells9010202

**Published:** 2020-01-14

**Authors:** Nicole Urban, Michael Schaefer

**Affiliations:** Leipzig University, Medical Faculty, Rudolf Boehm Institute of Pharmacology and Toxicology, Härtelstraße 16-18, 04107 Leipzig, Germany; nicole.urban@medizin.uni-leipzig.de

**Keywords:** transient receptor potential canonical, *Artemisia annua*, secondary plant metabolites, high-throughput compound screening, membrane-delimited activator, non-selective cation channel, intracellular calcium homeostasis

## Abstract

(1) Background: Members of the TRPC3/TRPC6/TRPC7 subfamily of canonical transient receptor potential (TRP) channels share an amino acid similarity of more than 80% and can form heteromeric channel complexes. They are directly gated by diacylglycerols in a protein kinase C-independent manner. To assess TRPC3 channel functions without concomitant protein kinase C activation, direct activators are highly desirable. (2) Methods: By screening 2000 bioactive compounds in a Ca^2+^ influx assay, we identified artemisinin as a TRPC3 activator. Validation and characterization of the hit was performed by applying fluorometric Ca^2+^ influx assays and electrophysiological patch-clamp experiments in heterologously or endogenously TRPC3-expressing cells. (3) Results: Artemisinin elicited Ca^2+^ entry through TRPC3 or heteromeric TRPC3:TRPC6 channels, but did not or only weakly activated TRPC6 and TRPC7. Electrophysiological recordings confirmed the reversible and repeatable TRPC3 activation by artemisinin that was inhibited by established TRPC3 channel blockers. Rectification properties and reversal potentials were similar to those observed after stimulation with a diacylglycerol mimic, indicating that artemisinin induces a similar active state as the physiological activator. In rat pheochromocytoma PC12 cells that endogenously express TRPC3, artemisinin induced a Ca^2+^ influx and TRPC3-like currents. (4) Conclusions: Our findings identify artemisinin as a new biologically active entity to activate recombinant or native TRPC3-bearing channel complexes in a membrane-confined fashion.

## 1. Introduction

Among mammalian transient receptor potential (TRP) channels, the “canonical” or “classical” TRPC3 isoform was the first to become functionally characterized upon heterologous expression [1]. Its direct activation by diacylglycerols added a new facet to the spectrum of actions caused by the membrane-resident lipid second messenger [2]. There are numerous functions of TRPC3 that have been deduced from the phenotype of TRPC3-deficient mice. One of the most striking effects is an impaired motor coordination that is linked to the strong expression of TRPC3 and its activation by phospholipase C (PLC)-coupled metabotropic glutamate receptors in a specific pool of cerebellar Purkinje neurons [3,4] and was confirmed by a similar effect in a mutant “moonwalker” mouse strain that carries a point mutation in the TRPC3-encoding gene [5]. Despite these and other data demonstrating that TRPC3 mediates a plethora of effects in the context of G-protein- or receptor tyrosine kinase-mediated signalling, its validation as a pharmacological target is still ongoing [6].

One of the limiting facts was the lack of a direct and isotype-specific activator. Large efforts have been undertaken to identify TRPC3 activators that avoid activation of protein kinase C. Among them, the benzoimidazole compounds GSK1702934A or BI-2 [7,8,9], and pyrazolopyrimidines [10] are the most potent and effective ones. While GSK1702934A is a combined activator of all three members of the TRPC3/TRPC6/TRPC7 subfamily, one of the pyrazolopyrimidines (named 4n in [10]) exhibits a substantial preference in activating TRPC3 over TRPC6.

In an attempt to identify novel modulators of TRPC3 channels, we screened a library of 2000 biologically active compounds (Spectrum Collection; MS Discoveries) on a cell line that stably expresses human TRPC3 C-terminally fused to a yellow fluorescent protein. The antimalarial drug artemisinin was the most effective TRPC3-activating hit that emerged from this screen. Validation and characterization of the sesquiterpene plant metabolite by complementary methods revealed an unexpected TRPC3-selective, PLC- and diacylglycerol-independent mode of action that was confirmed in rat pheochromocytoma cells that endogenously express TRPC3 and TRPC6 [11].

## 2. Materials and Methods

### 2.1. Chemicals

Artemisinin and 1-oleoyl-2-acetyl-*sn*-glycerol (OAG) were purchased from Sigma Aldrich (Munich, Germany). All other artemisinin derivatives (artemether, artenimol, arteether, and artesunate) were from Santa Cruz Biotechnology (Heidelberg, Germany). GSK1702934A and GSK-417651A were obtained from Tebubio (Offenbach, Germany), SAR7334 hydrochloride was from MedChemexpress (Monmouth Junction, NJ, U.S.A.), and CBN 2910-0498 and BTD were from ChemDiv (San Diego, CA, U.S.A.). All other reagents were of analytical grade.

### 2.2. Cell Culture

Human embryonic kidney 293 (HEK293) cells were stably transfected with expression plasmids that encode hTRPC3-YFP, hTRPC6-YFP, hTRPC7-YFP, mTRPC4ß-YFP, rTRPV1-CFP, rTRPV2-YFP, rTRPV3-YFP, mTRPV4-YFP, or hTRPM2 as previously described. Cell lines were cultured in Earle’s minimum essential medium (MEM; Sigma, Munich, Germany), supplemented with 10% fetal calf serum (Gibco Thermo Fisher Scientific, Darmstadt, Germany), 2 mM l-glutamine, and required concentrations of geneticin in a range of 0.4–1 mg/mL. A HEK293_mTRPC5-YFP_ cell line was maintained and induced as described before [12]. The HEK293_mTRPM3α2_ cells (expressing the Ca^2+^-permeable main variant of mouse TRPM3) were kindly provided by Johannes Oberwinkler (Marburg, Germany), the HEK293_hTRPA1_ cells that constitutively overexpress the human TRPA1 were from Dr. Natalie Tigue and Dr. Steve Moore (GlaxoSmithKline, Harlow, UK). With the exception of mTRPC5-YFP, all cell lines constitutively overexpress the respective TRP channel under the control of a cytomegalovirus (CMV) promoter. A HEK cell line stably expressing the human protein kinase C (PKC) ε C-terminally tagged with YFP [13] was generated and maintained in the same medium as HEK_TRPC3-YFP_ cells. PC12 cells were cultured in RPMI-1640 (Sigma, Munich, Germany) with 10% fetal calf serum, 2 mM l-glutamine, 100 U/mL penicillin, and 0.1 mg/mL streptomycin. For electrophysiological and single cell [Ca^2+^]_i_ imaging experiments, PC12 cells were plated onto 25 mm poly-l-lysine-coated glass coverslips and measured 24–48 h after seeding. All cells were maintained at 37 °C in a 5% CO_2_-aerated, humidified atmosphere.

### 2.3. Detection of Cell Viability

To assess cytotoxicity of artemisinin and its derivatives, a 3-(4,5-dimethylthiazol-2-yl)-2,5-diphenyltetrazolium bromide (MTT) assay in parental HEK293 cells was carried out. Cells were seeded in poly-l-lysine-coated 96-well plates at a density of 10,000 cells per well. After 24 h, the medium was exchanged, and compounds or the corresponding solvent were added and incubated for another 24 h. Then, the medium was removed, and a fresh medium containing 0.5 mg/mL MTT was added. After 3 h of incubation, supernatants were aspirated, and cells containing formazan crystals were lysed with 100% DMSO. Finally, absorbance of DMSO-dissolved formazan was measured at 560 and 670 nm with a plate reader (Polarstar Omega, BMG Labtech, Germany). The differences of both extinctions were calculated, and values were normalized to a solvent control (0.25% DMSO).

### 2.4. Fluorometric [Ca^2+^]_i_ Imaging

All fluorometric Ca^2+^ assays were done in (4-(2-hydroxyethyl)-1-piperazineethanesulfonic acid) (HEPES)-buffered saline (HBS), containing 132 mM NaCl, 6 mM KCl, 1 mM MgCl_2_, 1 mM CaCl_2_, 5.5 mM d-glucose, 10 mM HEPES, and 0.035% bovine serum albumin (BSA) adjusted to pH 7.4 with NaOH. For measurements in Ca^2+^-free bath solutions, a modified HBS without CaCl_2_ was supplemented with 200 µM ethylene glycol-bis(β-aminoethyl ether)-*N*,*N*,*N*′,*N*′-tetraacetic acid (EGTA).

The primary compound screen and the generation of concentration response curves in HEK293 cells or in PC12 cells were performed by using a custom-made fluorescence plate imager built into a robotic liquid handling station equipped with a 96-tip multichannel head (Freedom Evo 150, Tecan, Männedorf, Switzerland) as described previously [14]. To this end, trypsinized cell suspensions were loaded with 4 µM fluo-4/AM (Invitrogen, Thermo Fisher Scientific, Waltham, MA, U.S.A.) in cell culture medium for 30 min at 37 °C. After brief centrifugation, cells were resuspended in HBS and dispensed into black pigmented, clear-bottom 384-well plates (Greiner µClear, Frickenhausen, Germany). Plates were mounted on the imager, and fluorescence signals were recorded with a cooled Zyla 5.5 camera (Andor, Belfast, UK) through a 515 nm long pass filter. The image acquisition was controlled by the µManager software (http://micro-manager.org) [15], and excitation with a LED array (470 nm) was synchronized by the camera trigger output. After baseline recording for about 60 s, the compound libraries or serial dilutions of activators or inhibitors were added to the cells by the liquid handling device. When inhibitory potencies were evaluated, a second pipetting step with the respective reference agonist was performed. Fluorescence intensities were calculated from image stacks for each well with ImageJ (National Institutes of Health, Bethesda, MD, U.S.A.) [16], corrected for background signals, and normalized to the initial intensities (F/F_0_). Concentration response curves were generated by fitting the data to a four-parameter Hill equation to obtain the E_min_, E_max_, EC_50_ (or IC_50_), and the Hill coefficient *n*.

The microfluorometric single-cell [Ca^2+^]_i_ analysis was performed on an inverted epifluorescence microscope (Carl Zeiss, Jena, Germany) and calibrated as described [14,17]. Cells were grown on 25 mm coverslips and loaded with 4 µM fura-2/AM (AAT Bioquest, Sunnyvale, CA, U.S.A.) in HBS containing 0.2% BSA for 30 min at 37 °C. After gently rinsing the coverslips with HBS, coverslips were mounted in a bath chamber and superfused with HBS containing 0.035% BSA.

### 2.5. Immunofluorescence Analysis

PC12 cells were seeded onto poly-l-lysine-coated glass coverslips, cultured for 2 d, and finally fixed with methanol, followed by combined permeabilization and blocking with phosphate-buffered saline (PBS), supplemented with 1% BSA, 0.1% Triton-X100 and 10% goat serum for one hour. The primary antibodies, polyclonal rabbit anti-TRPC3 (Alomone Labs, Jerusalem, Israel; ACC-016; 1:100) and monoclonal mouse anti-tyrosine hydroxylase (Merck, Darmstadt, Germany; MAB 5280; 1:800), were incubated overnight. After washing samples three times for 5 min in PBS, secondary antibody decoration with Alexa Fluor 488-conjugated goat anti-rabbit (Invitrogen; Eugene, OR, U.S.A.; A11008; 5 µg/mL) and Cy3-conjugated donkey anti-mouse antibodies (Jackson ImmunoResearch Laboratories, Cambridgeshire, U.K.; 1:400) was done for 2 h. Finally, coverslips were washed again and mounted with Fluoromount-G (VWR International GmbH, Darmstadt, Germany), containing 4′,6-diamidino-2-phenylindole (DAPI) to counterstain nuclei of PC12 cells. Fluorescence detection was performed with a Leica DMi8 confocal microscope using an HC PL APO CS2 63×/1.4 oil objective and the LAS X software (Leica Microsystems, Wetzlar, Germany).

### 2.6. Electrophysiological Procedures

The recordings of TRPC3, TRPC6, or TRPC7 membrane currents in HEK293 or PC12 cells were realized by using a Multiclamp 700B amplifier with a Digidata 1440A digitizer controlled by the PClamp 10 software (all Molecular Devices, Sunnyvale, CA, U.S.A.). Recordings were obtained at room temperature either in the whole-cell or in an outside-out configuration as indicated. Cells were seeded as described above, but at lower density, mounted in a chamber (Warner Instruments, Hamden, CT; U.S.A.), placed onto the stage of an inverted microscope, and perfused with required solution. The standard extracellular solution contained 140 mM NaCl, 5 mM CsCl, 2 mM MgCl_2_, 1 mM CaCl_2_, 10 mM HEPES, and 0.035% BSA adjusted to pH 7.4 with NaOH. The composition of standard intracellular solution was 140 mM CsCl, 1 mM MgCl_2_, 5 mM EGTA, and 10 mM HEPES adjusted to pH 7.25 with CsOH; the osmolarity was 295 mOsm l^−1^. In outside-out measurements, the pipette solution contained 5 mM EGTA and 1.1 mM CaCl_2_ to achieve a calculated free Ca^2+^ concentration of 100 nM.

Patch pipettes were pulled from borosilicate glass capillaries BG150F-8P (Science Products, Hofheim, Germany) with a PIP6 pipette puller (HEKA Elektronik, Lambrecht, Germany). They had a resistance of 4–10 MΩ when filled with the respective pipette solution. In all whole-cell measurements, the series resistance was lower than 13 MΩ, and compensated by 70%. If the access was unstable during the experiment (increase in series resistance by more than 15% at the end of the experiment), the cell was omitted from the analysis. To record current/voltage (I/V) curves, voltage ramps (400 mV s^−1^) ranging from –100 to +100 mV were applied. Currents were filtered at 3 kHz with a four-pole Bessel filter and sampled at 10 kHz.

### 2.7. Statistical Analyses

Concentration response functions were constructed by nonlinear curve fitting of Hill equations to the experimental data, applying the iterative Solver function of MS Excel (Microsoft, Redmond, WA, USA). For comparing two groups, Student´s t-test was applied either with paired datasets, to analyze the impact of modulators on current densities or fluorescence intensities in single cells, or with an unpaired t-test in all other cases. Multiple comparisons were statistically analyzed by one-way ANOVA with a Dunn–Sidak post-hoc test (OriginPro, Northampton, MA, USA). *p* < 0.05 was accepted as significant.

## 3. Results

### 3.1. Primary Screening, Hit Validation, and Concentration–Response Analyses

**Figure 1:**
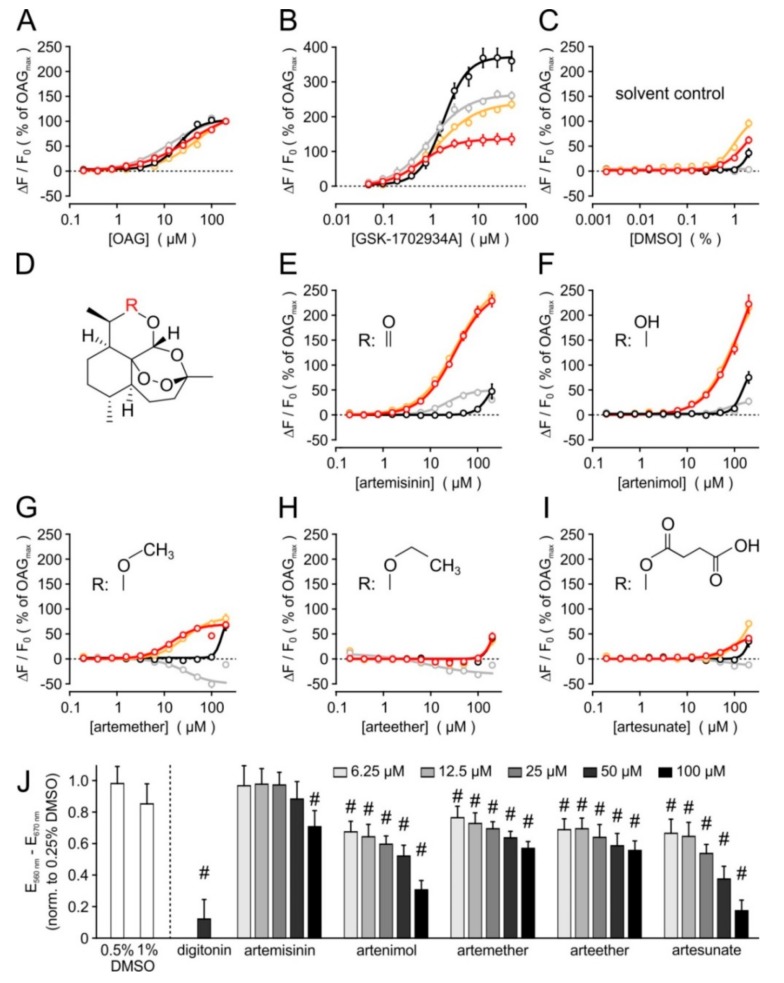
Concentration response analysis of known and novel TRPC3/TRPC6/TRPC7 activators, and cytotoxicity analysis. The potency and efficacy of various compounds to elicit a [Ca^2+^]_i_ response was tested in fluo-4-loaded cell suspensions, applying HEK293 cells that were stably transfected with cDNA plasmids encoding either TRPC3 (red symbols and lines), TRPC6 (black), or TRPC7 (grey). HEK cells that form TRPC3:TRPC6 heteromeric complexes by stably co-expressing TRPC3 and TRPC6 are also included (orange). Concentration–response curves for the indicated known activators (**A**,**B**), and for artemisinin and related compounds (**E**–**I**) are shown. Since 10 mM stock solutions were prepared for all compounds, the corresponding solvent control (**C**) applies to all other data. The common substructure for artemisinin-related compounds (**D**) with the respective substituents R (insets in **E**–**I**) are depicted. Data are aggregated from 4–6 independent experiments performed in duplicate, each, and displayed as means ± S.E. after normalizing the data to the responses elicited by 200 µM OAG at the same day. (**J**) Artemisinin and related compounds were added to parental HEK293 cells in growth medium at the indicated concentrations, incubated for 24 h, and the metabolic activity was assayed with an MTT test. Solvent controls (DMSO) and a positive control (50 µM digitonin) were included, and data were expressed after normalisation to the respective 0.25% DMSO controls. Data represent means ± S.D. of 5–8 experiments; #: *p* < 0.05.

To identify direct activators of TRPC3 channels, we performed a medium-throughput screen, applying a compound library consisting of 2000 biologically active drugs, molecularly defined natural products, signalling pathway modulators, and toxins. Upon acute addition of the compounds at a concentration of 20 µM to fluo-4-loaded HEK cells that stably expressed a TRPC3-YFP fusion protein (HEK_TRPC3-YFP_), an immediate and transient increase in the fluorescence signal of the Ca^2+^ indicator became apparent in wells that contained artemisinin and artenimol (Figure S1A). A counterscreen with cells expressing the closely related TRPC6 channel showed no signals in these wells. The transient kinetics of fluorescence intensities hinted towards a channel activation rather than a toxic effect or a fluorescence of the compounds. An initial validation was obtained after “cherry picking” by reassessing the effects of artenimol and artemisinin in HEK_TRPC3-YFP_ cells, but also in un-transfected parental HEK293 cells, which showed no response to either compound at concentrations up to 50 µM (Figure S1B–E and Figure S2A,B). The complete results of the primary screening and initial hit validation are summarized in Table S1. In a secondary screen, comprising concentration response analyses of artemisinin and arteminol (but also the related compounds artemether, arteether, and artesunate), the biological activity to activate Ca^2+^ entry into TRPC3-expressing cells in a concentration-dependent manner was confirmed (Figure 1). In contrast to the well-established mixed TRPC3/TRPC6/TRPC7 activators 1,2-oleoyl-acetyl-*sn*-glycerol or GSK-1702934A, however, artemisinin and artenimol displayed a marked preference to activate TRPC3, but not the closely related TRPC6 channel. TRPC7 was weakly activated by artemisinin, remained largely unaffected by artenimol, and was inhibited by artemether or arteether. The potency to activate Ca^2+^ entry through TRPC3 was 32.8 µM for artemisinin and 14.8 µM for artemether. The TRPC3 activation by up to 200 µM arteminol did not reach saturation, and arteether or artesunate did not exert signals that were significantly stronger than responses to the solvent (DMSO, see Figure 1C,H,I). Of note, HEK293 cells that were stably transfected with both TRPC3 and TRPC6 and presumably form heteromeric TRPC3:TRPC6 channel complexes responded to artemininin, artenimol, or artemether in a very similar fashion as HEK_TRPC3-YFP_ cells (orange symbols and lines in Figure 1E–G).

### 3.2. Selectivity Profiling and Analysis of Cytotoxicity

An extended selectivity profiling of artemisinin was generated by measuring [Ca^2+^]_i_ responses in a panel of stably transfected HEK293 cell lines that overexpressed TRPC4, TRPC5, TRPA1, TRPM2, TRPM3, TRPM8, TRPV1, TRPV2, TRPV4, or TRPV4. With the exception of the poorly specific irritant sensor TRPA1, none of the cell lines responded to the addition of 100 µM artemisinin with significant increases in [Ca^2+^]_i_, whereas the subsequent stimulation with the respective channel-specific activators was still effective (Figure S3). Notably, we also did not observe a significant inhibition of the investigated channels by artemisinin compared to the solvent control, but only a slightly increased response of TRPV2-expressing cells to 2-aminoethoxydiphenyl borate (2-APB; final concentration: 300 µM). The unexpected subtype selectivity of artemisinin and related compounds prompted us to investigate the properties of these new TRPC3 activators in more detail. In an MTT test, exposure of parental HEK293 cells to artemisinin for 24 h did not reduce the metabolic activity at concentrations up to 50 µM. A slight and statistically significant reduction of metabolic activity was only seen in the presence of 100 µM artemisinin (Figure 1J). Since the other compounds displayed stronger cytotoxic effects, and since artemisinin seemed to exert the highest efficacy to activate Ca^2+^ entry through TRPC3, we focused on artemisinin for all following experiments.

**Figure 2:**
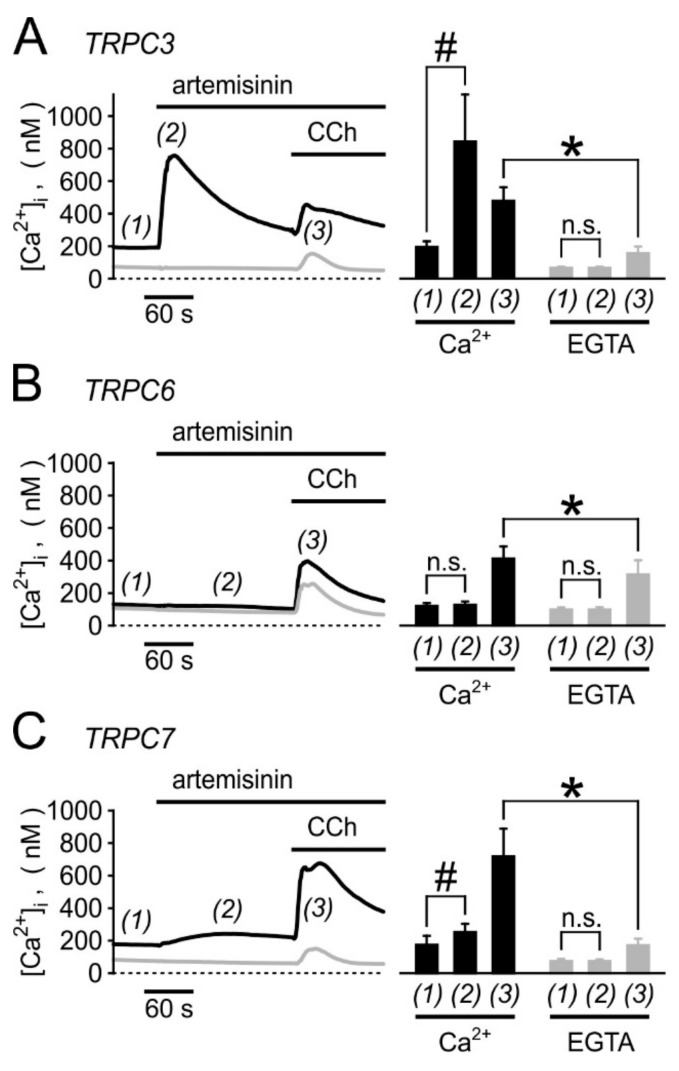
Microfluorometric single-cell analysis of artemisinin-induced increases in [Ca^2+^]_i_ in fura-2-loaded HEK cells stably expressing TRPC3 (**A**), TRPC6 (**B**), or TRPC7 (**C**). Calibrated [Ca^2+^]_i_ imaging analysis was performed in cells that overexpressed the indicated TRPC channel during sequential addition of 50 µM artemisinin and 1 mM carbachol (CCh). Experiments were performed either in standard bath solution, containing 1 mM Ca^2+^ (black lines and bars), or in a nominally Ca^2+^-free HBS buffer, supplemented with 200 µM EGTA (grey lines and bars). Time courses of averaged responses (left), and statistical analysis of 6–11 independent imaging experiments with means and S.D. (right) are shown. Significant differences (*p* < 0.05, Student´s *t*-test with paired data: #, with unpaired data: *) are marked for the indicated comparisons; n.s.: difference is statistically not significant.

Calibrated microfluorometric single-cell [Ca^2+^]_i_ analyses in fura 2-loaded cells confirmed the responses in TRPC3-overexpressing HEK cells, while TRPC6-expressing cells remained unaffected and TRPC7-overexpressing cells showed only a weak [Ca^2+^]_i_ signal (Figure 2; *n* = 6–11 experiments). In Ca^2+^-free bath solutions (nominally Ca^2+^-free HBS supplemented with 200 µM EGTA), the signals in TRPC3- or TRPC7-expressing cells were abolished, while Ca^2+^ mobilisation from intracellular stores by stimulating a muscarinic receptor with 1 mM carbachol remained detectable. We therefore conclude that artemisinin indeed triggers a Ca^2+^ influx through TRPC3 rather than a mobilisation from internal stores, which would indicate an involvement of PLC. Further evidence for a PLC-independent mode of action came from the observation that artemisinin does not induce [Ca^2+^]_i_ signals in parental HEK293 cells, while a [Ca^2+^]_i_ response to a subsequent stimulation with carbachol remained unchanged (Figure S2A–C). Finally, 50 µM artemisinin did not induce a discernible plasma membrane translocation of a diacylglycerol-sensitive PKCε-YFP fusion protein (Figure S2D,E). From these data, we conclude that artemisinin activates TRPC3 in a PLC-independent manner.

In fura 2-loaded, transfected HEK293 cells that transiently overexpress the mouse TRPC3 ortholog, the basal [Ca^2+^]_i_ was already elevated to 311 ± 134 nM (mean and S.D., *n* = 215 single cells measured in two independent experiments), but 50 µM artemisinin induced a further increase to 1140 ± 635 nM. Thus, the artemisinin-induced TRPC3 activation is not restricted to the human TRPC3 ortholog.

### 3.3. Synergism of Artemisinin and Diacylglycerol Effects on TRPC3

When sequentially applied after a challenge with maximally effective artemisinin concentrations, 100 µM OAG failed to induce a further increase in [Ca^2+^]_i_ in microfluorometric single cell [Ca^2+^]_i_ imaging experiments on HEK_TRPC3-YFP_ cells or in HEK cells that were transiently transfected to express mouse TRPC3 (data not shown). Likewise, artemisinin did not induce stronger Ca^2+^ influx signals in HEK_TRPC3-YFP_ cell suspensions after a preincubation with 500 nM, 1 µM, or 5 µM OAG. Finally, a preincubation with the DAG kinase inhibitor R59949 (10 µM for 5 min), or a DAG lipase inhibitor RHC 80267 (10 µM for 5 min), did not increase the responses to the acute stimulation with various artemisinin concentrations (1–200 µM). From these data, we concluded that artemisinin effects do not critically rely on the availability of diacylglycerols.

**Figure 3:**
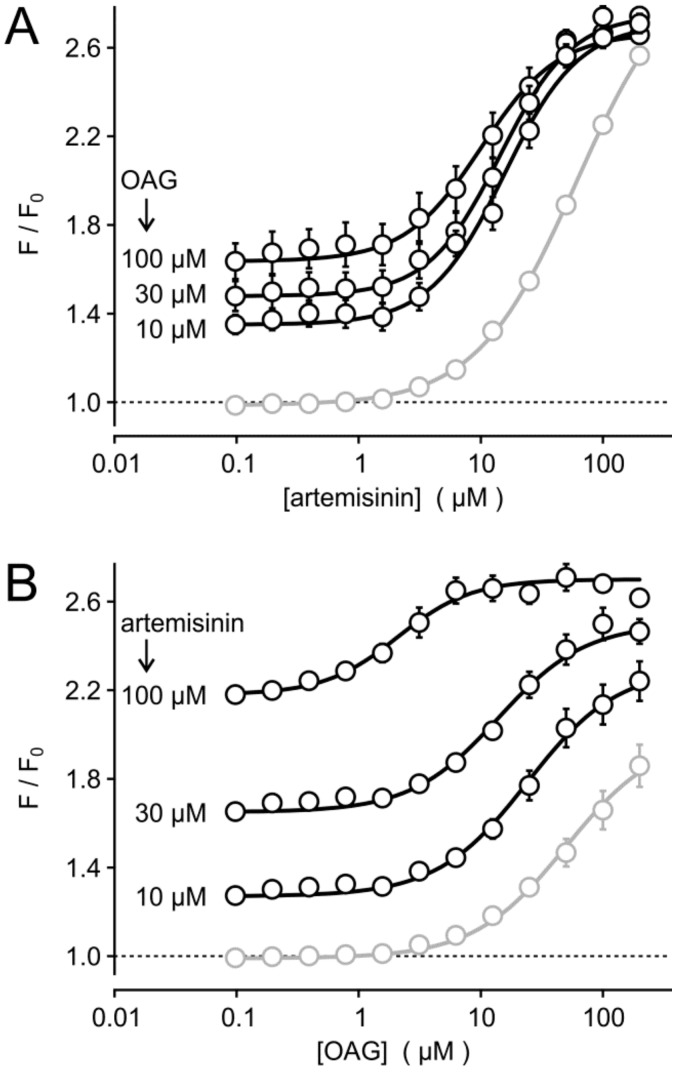
Effects of acute co-stimulation of HEK_TRPC3-YFP_ cells with various concentrations of artemisinin and OAG. Fluo 4-loaded HEK_TRPC3-YFP_ cell suspensions were assayed in a fluorescence imaging plate reader during acute co-application of serially diluted artemisinin without (grey symbols and lines) or with OAG added at the indicated fixed concentrations (**A**). (**B**) Similar experiment as in (**A**), but with serially diluted OAG in the absence or presence of the indicated fixed artemisinin concentrations. Data represent means and S.E. (*n* = 4 in two independent experiments).

Interestingly, the acute co-stimulation with various artemisinin and OAG concentrations in fluo 4-loaded HEK_TRPC3-YFP_ suspensions confirmed that the half maximally effective concentration (EC_50_) of artemisinin was not affected by the simultaneous application of 10–100 µM OAG (Figure 3A), but the EC_50_ of OAG was shifted to lower concentrations in the presence of 30 or 100 µM artemisinin (Figure 3B). This effect may hint to a positive, allosteric effect of artemisinin by sensitizing TRPC3 to lower OAG concentrations. One should, however, also consider that OAG is poorly soluble without carriers (e.g., DMSO, bovine serum albumin, nonionic detergents). Thus, sensitization may be mimicked by the physicochemical properties of artemisinin, which is also poorly water soluble. Taken together, artemisinin seems to be effective without additional diacylglycerols, but a response-enhancing effect to diacylglycerols cannot be excluded.

### 3.4. Electrophysiological Analysis

**Figure 4:**
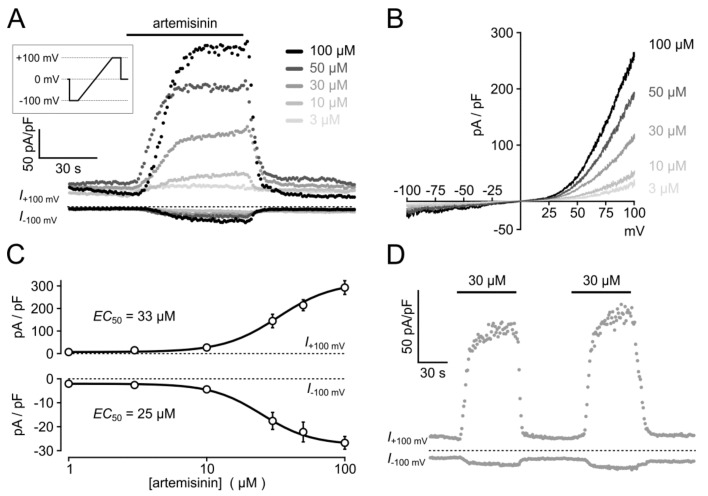
Electrophysiological analysis of artemisinin-induced whole-cell currents in HEK_TRPC3-YFP_ cells. The voltage protocol depicted in the box (duration of 0.8 s) was applied in 1 s intervals, and steady state inward and outward current densities averaged at potentials of –100 or +100 mV as well as voltage ramps (0.4 Vs^−1^) were extracted from the data. (**A**) Current densities at +100 and –100 mV, respectively. Data resulting from the application of the indicated concentrations of artemisinin, followed by a wash-out of the activator, are overlaid. (**B**) Typical examples of I/V curves, recorded close to the respective current peaks in cells exposed to the indicated artemisinin concentrations. (**C**) Outward and inward current densities were measured in 12–27 cells for each artemisinin concentration, as shown in (**A**), and aggregated to construct concentration–response curves. The indicated EC_50_ values for artemisinin-induced outward and inward currents were obtained by fitting a four-parameter Hill equation to the data (respective Hill coefficients: 2.2 and 2.4). (**D**) Example of ionic currents elicited during repeated application of 30 µM artemisinin to a HEK_TRPC3-YFP_ cell.

**Figure 5:**
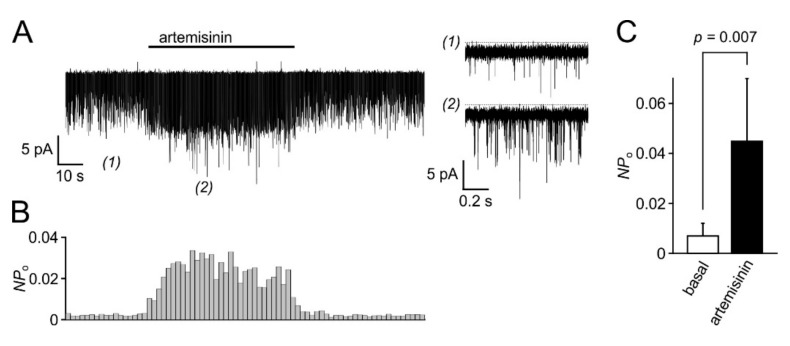
Channel activity induced by artemisinin in outside-out patches taken from HEK_TRPC3-YFP_ cells. Outside-out patches were obtained from HEK_TRPC3-YFP_ cells, and ionic currents were continuously recorded at a patch potential of –100 mV. Artemisinin (50 µM) was applied to the bath chamber as indicated by the bar (**A**, left). Short episodes before (1) and during application of artemisinin (2) are re-plotted at higher temporal resolution (right). Note that unitary current amplitudes are hardly discernible due to the very short channel opening events of TRPC3 channels. (**B**) The open probability (*P*_o_) for the unknown number *N* of channels in the patch of the experiment shown in (**A**) was calculated over 2 s bins and plotted at the same time scale. (**C**) Aggregated data (means and S.D.) of peak *NP*_O_ values obtained as shown in (**B**) were obtained from 6 isolated outside-out patches before (basal) and during application of 50 µM artemisinin.

**Figure 6:**
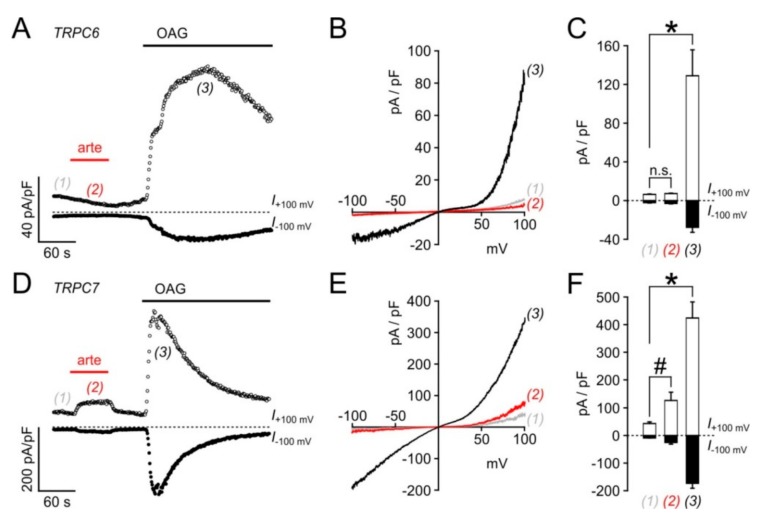
Effects of artemisinin on ionic currents through TRPC6 and TRPC7. Whole-cell current densities were measured in HEK_TRPC6-YFP_ (**A**–**C**) and in HEK_TRPC7-YFP_ cells (**D**–**F**) in a similar fashion as shown in Figure 4A, during application and wash-out of 100 µM artemisinin (arte). To ascertain that the cells expressed the respective channel, cells were subsequently exposed to 50 µM OAG as indicated by the black bars (**A**,**D**). (**B**,**E**) Current densities during voltage ramps (ranging from −100 to + 100 mV at 400 mV s^−1^) taken at the indicated time points from experiments shown in (**A**,**D**). (**C**,**F**) Current densities before (1), and during (2) exposure to 100 µM artemisinin, and peak current densities reached during stimulation with 50 µM OAG (3) were recorded in 9 cells, each, and depicted as means and S.E. Statistically significant differences were denoted as * and # for the indicated comparisons (*p* < 0.05; Student´s *t*-test with paired data); n.s.: statistically not significant.

In electrophysiological recordings taken from HEK_TRPC3-YFP_ cells in the whole cell configuration, the acute addition of artemisinin elicited significant increases in outward and inward current densities that were measured at +100 and −100 mV, respectively (Figure 4A). The current increases were rapidly reversible upon wash-out of the activator. In current voltage analyses, applying voltage ramps between –100 to +100 mV (400 mV/s), a typical outwardly rectifying current was detected (Figure 4B). The EC_50_ of artemisinin to activate TRPC3 currents was 33 µM for outward currents and 25 µM for inward currents (Figure 4C; *n* = 12–27 cells), and a repeated application of the activator caused corresponding increases in inward and outward current densities with no signs of channel rundown (Figure 4D). In excised outside-out membrane patches, the addition of 50 µM artemisinin was associated with an about 5.5-fold increase in the open probability (*n* = 6) with short-lived single-channel events that are characteristic for TRPC3 (Figure 5). Again, the channel activity was reversible upon wash-out of artemisinin. Ionic currents in TRPC6- or TRPC7-expressing HEK cells challenged with 100 µM artemisinin remained unaffected (TRPC6) or were only weakly enhanced (TRPC7), whereas a subsequent exposure to 50 µM OAG caused increases in current densities with current–voltage relationships that are typical for TRPC6 and TRPC7 (Figure 6).

### 3.5. Biological Activity Towards Native TRPC3-Bearing Channel Complexes

**Figure 7:**
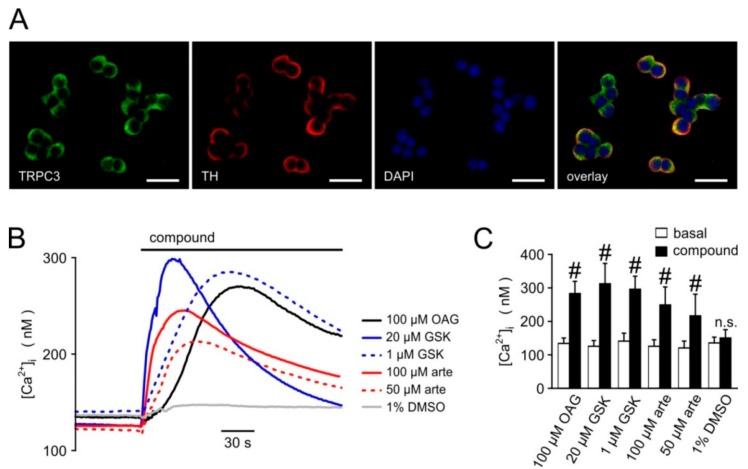
Expression of TRPC3 and functional responses to TRPC activators in PC12 rat pheochromocytoma cells. (**A**) Immunofluorescence analysis of TRPC3 (green channel) and tyrosine hydroxylase (TH; red channel) expression in PC12 cells. Nuclei were counterstained with DAPI (blue channel). Bars: 20 µm scale. Time course of the [Ca^2+^]_i_ (**B**) and averaged basal and peak [Ca^2+^]_i_ values (**C**) were recorded by microfluorometric analysis in fura-2-loaded PC12 cells grown on glass coverslips. The cells were challenged with the indicated concentrations of OAG, GSK-1702934A (GSK), and artemisinin (arte) or the final concentration of its solvent (DMSO). Data represent averages (**B**) or means and S.D. (**C**) of 6–12 imaging experiments for each compound. Significant increases compared to the respective basal values are indicated (#: *p* < 0.05; Student´s *t*-test for paired data).

**Figure 8:**
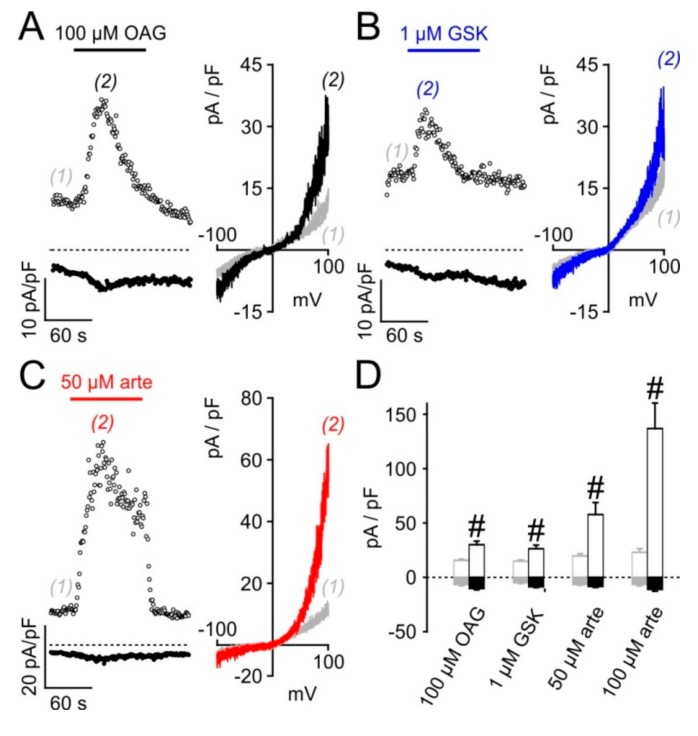
Electrophysiological whole-cell recordings of artemisinin-induced TRPC3-like currents in PC12 cells. Current densities in PC12 cells were recorded at +100 mV (upper traces) and –100 mV (lower traces) as described in Figure 4A during the application of 100 µM OAG (**A**), 1 µM GSK-1702934A (GSK; **B**), or 50 µM artemisinin (arte; **C**). Time courses of current densities (left panels) and I/V curves taken at the indicated time-points (right panels) are depicted. (D) Statistical analysis of outward (open bars) and inward current densities (filled bars) in 11–15 PC12 cells before (grey) and during the exposure to the indicated activators (black). #: statistically significant difference of both outward and inward current densities compared to the respective basal values.

**Figure 9:**
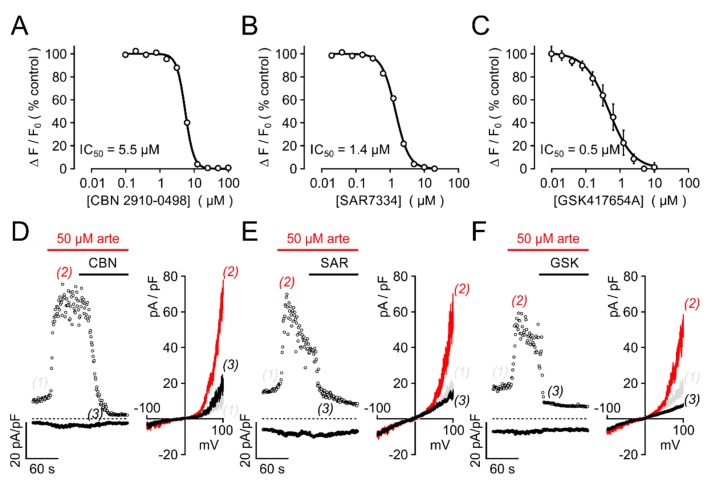
Artemisinin-induced ionic currents in HEK293 cells and in PC12 cells are sensitive to established TRPC3 inhibitors. (**A**–**C**) Serial dilutions of the indicated inhibitors were applied to HEK_TRPC3-YFP_ cells at the indicated final concentrations for 5 min, and responses to acute challenge with 50 µM artemisinin were measured in fluo-4-loaded HEK_TRPC3-YFP_ cell suspensions. Concentration–response curves were normalized to responses measured without an inhibitor (controls), and IC_50_ values were obtained by fitting a four-parameter Hill equation to the data. Data are compiled from 4–6 independent experiments performed in duplicate, each. (**D**–**F**) Ionic currents in PC12 cells were induced by 50 µM artemisinin and recorded as in Figure 7C, but with inhibitors CBN 2910-0948 (CBN, 10 µM; **D**), SAR7334 (SAR, 5 µM; **E**), or GSK417654A (GSK, 5 µM; **F**) added in the continuous presence of artemisinin. Note the markedly accelerated current decay during application of the inhibitors (left panels). Corresponding I/V curves taken at the indicated time points are plotted (right panels).

We next sought to investigate the effects of artemisinin on native TRPC3 channel complexes. In PC12 pheochromocytoma cells, expressions of TRPC3 and of tyrosine hydroxylase, a key enzyme to generate adrenergic neurotransmitters, were detectable by immunofluorescence (Figure 7A). Since anti-TRPC3 antibodies might suffer from a lack of specificity, we tested for functional responses of these cells to various established TRPC3 activators and artemisinin. In fura 2-loaded PC12 cells, significant increases in [Ca^2+^]_i_ were triggered with the TRPC3/6/7-overarching activators OAG and GSK-1702934A, but also with 50 or 100 µM artemisinin (Figure 7B; 6–12 experiments for each compound). Electrophysiological recordings in PC12 cells confirmed the activator-induced and reversible increase in outwardly rectifying current densities with a reversal potential close to 0 mV (Figure 8). Further proof for the functional expression of endogenous TRPC3 channel-bearing complexes was obtained by testing the inhibitor sensitivity. Artemisinin-induced increases in [Ca^2+^]_i_ in HEK_TRPC3-YFP_ cells were sensitive to several recently published TRPC3 inhibitors, including CBN 2910-0498, SAR7334, or GSK417654A with IC_50_ values that were in the same range as those reported for TRPC3 activation by OAG (Figure 9A–C). In PC12 cells, ionic currents that were stimulated by 50 µM artemisinin were rapidly reversed by adding either 20 µM CBN 2910-0498, 10 µM SAR7334, or 10 µM GSK417654A (Figure 9D–F).

## 4. Discussion

The activation of TRPC3 by artemisinin in excised membrane patches points to a membrane-confined mechanism of the drug either by directly binding to the channel protein or to a membrane-associated interaction partner of TRPC3. This assumption is further supported by the notion that neither artemisinin nor artenimol cause an increase in [Ca^2+^]_i_ in parental HEK cells. If artemisinin were activating PLC, an inositol-1,4,5-trisphosphate-mediated release of Ca^2+^ from the endoplasmic reticulum and no TRPC isoform-specificity would be expected. In addition, a fluorescent diacylglycerol-sensitive PKCε-YFP did not translocate to the plasma membrane when cells were exposed to 50 µM artemisinin.

The activation of TRPC3 by artemisinin gives rise to outwardly rectifying ionic currents that reverse close to 0 mV and display very short-lived single-channel openings in the outside-out patch configuration. Thus, it exhibits properties that are identical to those elicited either by membrane-permeable diacylglycerols or by activation of PLC-coupled receptors. The artemisinin-induced TRPC3 activation did not rely on a co-application of OAG, but the concentration dependence of OAG was shifted to lower concentrations when artemisinin was acutely co-applied. The activation of TRPC3 is surmountable by the established TRPC3 inhibitors CBN 2910-0498, SAR7334, or GSK417654A at concentrations that are reported for the inhibition of diacylglycerol- or receptor-induced TRPC3 activation. Therefore, we assume that artemisinin facilitates channel opening in a way that closely resembles the physiological activation mechanism, without changing the ion permeation selectivity and without locking TRPC3 in an open state. It should also be noted that artemisinin activates recombinant human and mouse TRPC3 or native TRPC3-bearing channel complexes in rat PC12 cells.

Direct activators are available for several PLC-dependent gated TRPC channels. Unfortunately, they often display little or no isoform specificity with regard to single members of the TRPC1/TRPC4/TRPC5 or TRPC3/TRPC6/TRPC7 subfamilies. By recruiting and activating protein kinase C and other C1 domain-bearing signalling proteins, membrane-permeable diacylglycerols are even more promiscuous with a coincident activation of channel-unrelated cellular signals. This promiscuity is shared by photoswitchable diacylglycerol mimics [18,19]. The compound GSK-1702934A indeed effectively and potently activates TRPC3 in a PLC-independent way, but it is a hybrid activator of all three members of the TRPC3/TRPC6/TRPC7 subfamily [7]. The same applies to a photoswitchable azobenzene derivative [20]. For TRPC6, hyperforin has been proposed as a direct activator [21], but more recent work raised serious doubts about a TRPC6-activating property of this herbal constituent, which instead seems to act as a protonophore [22]. Thus, a TRPC6-selective activator still does not exist, and positive allosteric modulators such as flufenamic acid or PAM20 are the only compounds to enhance TRPC6 activity in an isotype-selective manner [23,24], and no isotype-selective activator or positive allosteric is available for TRPC7 channels.

A potent and highly TRPC3-prevalent activator, 4n [10], has been published during our ongoing study. It seems to be a highly attractive compound, but is not freely available so far. Artemisinin, which is not only TRPC3-prevalent over TRPC6, but rather inhibits TRPC6, may fill this gap. In addition, the chemical structures of 4n and of artemisinin are unrelated. Thus, similar findings using two activators with unrelated structures would strengthen the pharmacological proof of TRPC3 being involved in an observed effect. Interestingly, and in contrast to 4n, the bicyclic central pharmacophore of the highly potent TRPC4/TRPC5 activator englerin A [25] and artemisinin share a common core structure with an epoxide-bridged seven-membered ring system. In addition, both TRPC-activating compounds are rich in hydrogen bond acceptors. A similarity screen within the DrugBank, using the spectrum walk algorithm of a Rchemcpp similarity search engine [26], and the englerin A core structure as a bait, forskolin, ginkgolides, and the artemisinin-derivative artemether were the first hits. It is, therefore, tempting to speculate that the pharmacophore may be shared across the TRPC subfamilies, where they may occupy related binding sites on their cognate targets.

The physicochemical properties of artemisinin with a calculated logP value of 3.11, no fixed charge, a polar surface area of 54 Å², and a relatively small molecular size (van-der-Waals volume of 261 Å³) are compatible with an unrestricted membrane permeability to access either intra- or extracellular sites of action. Thus, the biological activity of artemisinin to elicit TRPC3 channel openings in the outside-out mode upon application to the extracellular side of the membrane should not be interpreted as proof of an extracellular binding site.

Considering that the plasma concentrations of artemisinin reached in malaria treatment typically do not exceed 1 µM [27], the effect of the drug on TRPC3 is presumably not related to its antiplasmodial activity, and vice versa. The order of potencies of the artemisinin-related compounds towards TRPC3 also do not reconcile their antiplasmodial activity [28]. Thus, although the molecular target of the antimalarial activity of artemisinin is still not convincingly determined and may include a promiscuous action on several plasmodial proteins [29,30], the TRPC3-activating property may be considered an off-target effect, and its channel-activating property may serve as a cell biological tool in the first instance. The range of possible fields of use of artemisinin and mono- or dimeric derivatives has, however, extended to antitumor activities [31] or metabolic disorders [32]. Finally, artemisinin may become a scaffold for synthetic or semisynthetic variation in order to further optimize the potency of TRPC3-selective targeting.

The expression of TRPC3 in the rat PC12 pheochromocytoma cell line has been reported [11,33]. In accordance with Tesfai et al., we found that neuronal growth factor treatment, which promotes terminal differentiation of PC12 cells, led to a lower response to artemisinin. TRPC3 transcripts can be found in many tissues and cell types, but its absolute abundance is highest in the brain, the pituitary gland, and in smooth muscle tissues [34,35]. Although the neural origin of pheochromocytoma cells, including PC12, may explain the functional expression of TRPC3, the epinephrine-producing cells of the adrenal medulla in pigs do not express TRPC3 [36]. Consistent with this report, we also did not find functional TRPC3-related signals in freshly isolated mouse or rat adrenal medulla cells, or in human pheochromocytoma cells (data not shown). Thus, the functional expression of TRPC3 in healthy tissues other than brain may be more restricted than frequently assumed. This property seems to change in diseased conditions, where aberrant TRPC3 expression or function can contribute to the pathophysiology of ataxia [5,37], cardiac arrhythmias [7,38], cardiac hypertrophy [39,40,41], cardiac and renal fibrosis [42,43], to allergic airway disease [44,45], or to inflammatory hyperalgesia [46]. Here, artemisinin may immediately become helpful to differentiate between the functional significance of TRPC3 and TRPC6, which are frequently co-expressed, and to further validate TRPC3 as a potential pharmacological target.

## Supplementary Materials

The following are available online at https://www.mdpi.com/2073-4409/9/1/202/s1, Figure S1: Selectivity of artemisinin effects tested for various TRP channel isoforms.
