# Supplementary material for: Direct Activation of TRPC3 Channels by the Antimalarial Agent Artemisinin
Source: Cells. 2020 Jan 14;9(1):202. doi: 10.3390/cells9010202 (PMC7016953; doi:10.3390/cells9010202)
Supplement: Supplementary file 1 [file cells-09-00202-s001.pdf]

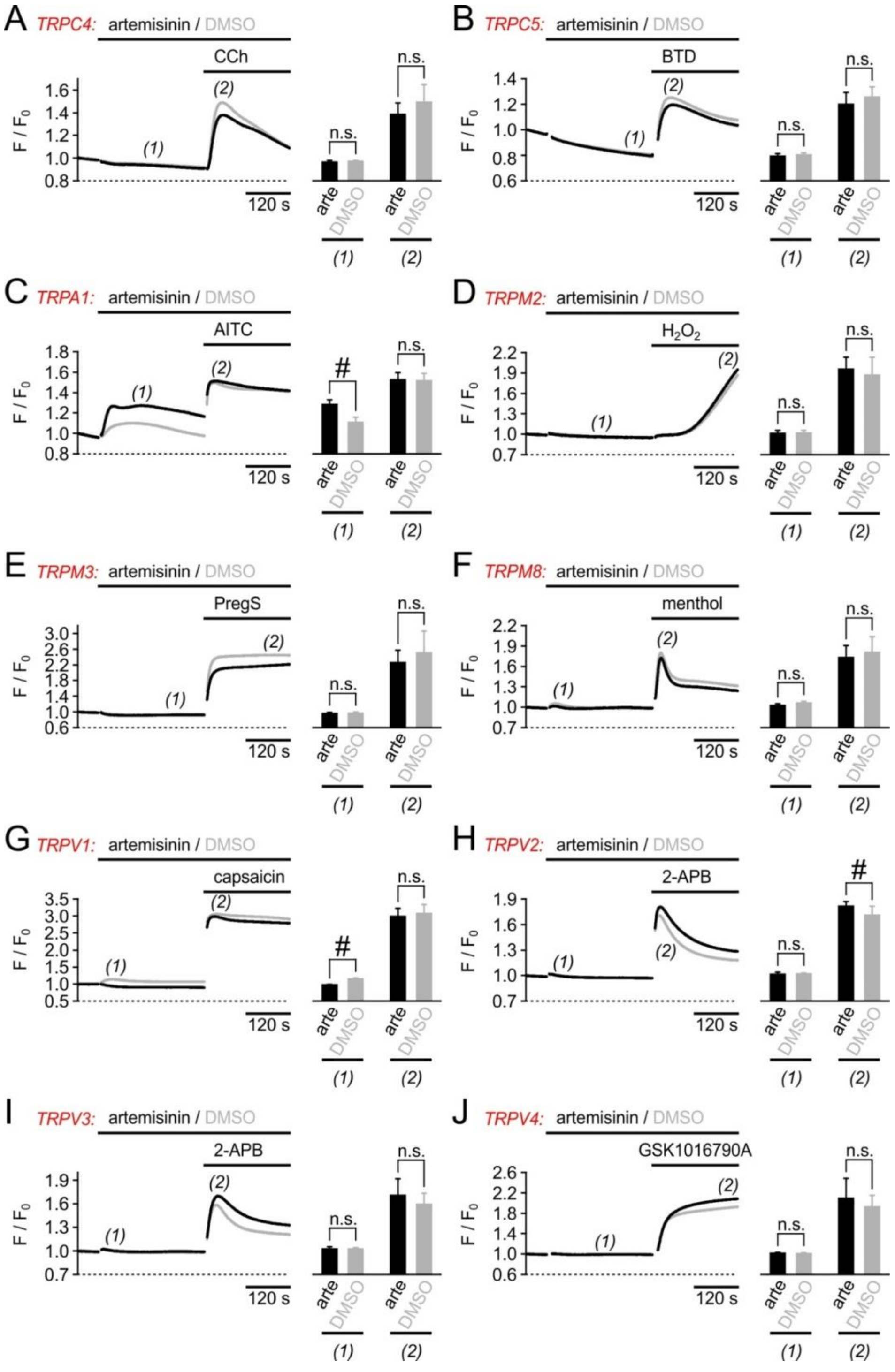

Figure S1. Selectivity of artemisinin effects tested for various TRP channel isoforms. Fluo-4-loaded suspensions of HEK293 cells overexpressing the indicated TRP channels were challenged with 100 μM artemisinin (black lines and bars), or 1% DMSO (grey lines and bars), followed by the addition of the respective TRP channel activators: 1 mM carbachol (CCh; TRPC4; 2.5 μM thapsigargin-pretreated cells), 50 μM of a TRPC5-activating benzothiadiazine (BTD; TRPC5), 100 μM allyl isothiocyanate (TRPA1), 1 mM H_2O_2 (TRPM2), 30 μM pregnenolone sulfate (PregS; TRPM3), 300 μM menthol (TRPM8), 2 μM capsaicin (TRPV1), 300 μM (TRPV2) or 30 μM (TRPV3) 2-aminoethoxydiphenyl borate (2-APB), and 100 nM GSK1016790A (TRPV4). Time courses (left panels), or means and S.D. of responses to artemisinin or DMSO (1) and the channel activators (2) determined in 4 experiments performed in duplicates, each, are shown. #: $p < 0.05$ for the indicated comparisons of unpaired data.
